# Review of functional markers for improving cooking, eating, and the nutritional qualities of rice

**DOI:** 10.3389/fpls.2015.00832

**Published:** 2015-10-14

**Authors:** Wendy C. P. Lau, Mohd Y. Rafii, Mohd R. Ismail, Adam Puteh, Mohammad A. Latif, Asfaliza Ramli

**Affiliations:** ^1^Department of Crop Science, Faculty of Agriculture, Universiti Putra MalaysiaSerdang, Malaysia; ^2^Laboratory of Food Crops, Institute of Tropical Agriculture, Universiti Putra MalaysiaSerdang, Malaysia; ^3^Bangladesh Rice Research InstituteGazipur, Bangladesh; ^4^Rice and Industrial Crops Research Centre, Malaysian Agricultural Research and Development InstituteSeberang Perai, Malaysia

**Keywords:** quantitative trait loci (QTL), DNA markers, rice quality, marker-assisted breeding (MAB), micronutrients, sequencing technology

## Abstract

After yield, quality is one of the most important aspects of rice breeding. Preference for rice quality varies among cultures and regions; therefore, rice breeders have to tailor the quality according to the preferences of local consumers. Rice quality assessment requires routine chemical analysis procedures. The advancement of molecular marker technology has revolutionized the strategy in breeding programs. The availability of rice genome sequences and the use of forward and reverse genetics approaches facilitate gene discovery and the deciphering of gene functions. A well-characterized gene is the basis for the development of functional markers, which play an important role in plant genotyping and, in particular, marker-assisted breeding. In addition, functional markers offer advantages that counteract the limitations of random DNA markers. Some functional markers have been applied in marker-assisted breeding programs and have successfully improved rice quality to meet local consumers’ preferences. Although functional markers offer a plethora of advantages over random genetic markers, the development and application of functional markers should be conducted with care. The decreasing cost of sequencing will enable more functional markers for rice quality improvement to be developed, and application of these markers in rice quality breeding programs is highly anticipated.

## Introduction

The important attributes of rice are its cooking and eating qualities, its phytochemicals and its micronutrients. The quality of rice needs to match the preferences of local consumers in order to be acceptable. Generally, Japanese people prefer short and sticky rice, whereas Indians prefer aromatic basmati rice which elongates when cooked. Furthermore, rice quality affects the market value, given that better quality rice is able to fetch a higher premium. Indian basmati rice and Thai jasmine rice are highly priced due to their distinctive aroma when cooked. The growing income and food diversification in Asian countries such as China (Sumner et al., 2001) and some European countries (Ferrero and Nguyen, 2004) have led consumers to prefer better quality rice.

While people in some parts of the world seek a better cooking and eating quality of their rice, people in other areas seek improved nutrition. Although micronutrients are only required in small quantities, they are necessary to maintain proper bodily function. In fact, two billion people worldwide suffer from micronutrient deficiencies, particularly in vitamin A, iodine, iron (Fe), and zinc (Zn) (World Health Organization, 2007). Therefore, research has been undertaken to increase the micronutrient content in rice to avert nutrient deficiency in the human diet, especially for populations where micronutrient deficiency is prevalent. Recently, Zn-biofortified rice has been developed to avert Zn deficiency in the diet of Bangladeshi people, particularly in children (Ahmad, 2013). The International Rice Research Institute (IRRI) is expected to release Fe-rich rice by the year 2029 to alleviate Fe deficiency anemia in needy countries (David, 2014).

Due to consumers’ demand for better rice quality, rice breeders all over the world are endeavoring to develop rice varieties with improved qualities that meet local demand. The quality of a rice variety is assessed after harvesting the grains from the plant. Prior to an assessment of the acceptability of the rice variety by panelists, the quality parameters of cooking and eating quality, and phytochemical and micronutrient composition are determined by using standard procedures (Dela Cruz and Khush, 2000). Determination of the quality parameters in each individual plant is laborious and time consuming. Certain chemical analyses might require large grain samples, which can be destructive to the plant material, especially during the early stage of breeding when the breeder’s seed supply is limited.

The advent of molecular marker technology in genetic analysis has revolutionized the research on rice quality. From the time scientists first ventured into using molecular markers, from the earliest protein markers to the current DNA markers, substantial effort in molecular mapping has identified chromosome regions carrying genes of interest. Undeniably, commonly used DNA markers, such as restriction fragment length polymorphism (RFLP), randomly amplified polymorphic DNA (RAPD), and simple sequence repeats (SSRs) have contributed to the mapping and association studies that led to the discovery of genes of interest. However, these DNA markers are derived randomly from polymorphic sites of the genome, and some can be located far from the gene of interest, which might be independent from the phenotype. Functional markers (FMs, also known as perfect markers) are an alternative to random DNA markers. FMs are developed from polymorphic sites within genes that cause phenotypic trait variation (Andersen and Lübberstedt, 2003). In contrast with random DNA markers, FMs are directly linked to the allele of the trait of interest (Varshney et al., 2005). Therefore, FMs are outcompeting random DNA markers, especially in marker-assisted breeding (MAB). Thus far, numerous FMs have been developed for the breeding of quality rice (**Table 1**), and some of them have been applied to breeding programs that have delivered desirable quality traits unambiguously (Yi et al., 2009; Jin et al., 2010).

**Table 1:** Candidate genes for Functional marker (FM) development and developed FM available for quality rice breeding.

Trait	Gene	Reference	Rice variety used	FM available	Method of FM identification
Fragrance	*Badh2*	Bradbury et al., 2005a	Sequence alignment of 14 fragrant on 64 non-fragrant varieties, mapping with F_2_ of Kyeema × Gulfmont	Bradbury et al., 2005b; Amarawathi et al., 2008, Shi et al., 2008; Sakthivel et al., 2009, Myint et al., 2012 Vanavichit et al., 2010	QTL mapping, map-based cloning and association studies


		Chen et al., 2008	Sequence alignment of 93-11 and Nipponbare		Transformation, RNAi, and comparing isogenic lines


		Shi et al., 2008	Sequence alignment of 24 fragrant rice varieties and ten non-fragrant varieties, mapping of F_2_ of Xiangjing02-5855 × Xiangxuenuo		
		Vanavichit et al., 2010	Sequence alignment of Khao Dawk Mali (KDML) and Nipponbare, Screening of F_6_ of KDML × Jao Hom Nin		
Amylose content (AC)	*Wx*	Ayres et al., 1997	Ninety-two US rice cultivars and breeding lines	Chen et al., 2010; Gao et al., 2012	Association studies
		Mikami et al., 2008	Near isogenic lines (NILs) of Taichung 65		
		Teng et al., 2012	Single segment substitution lines (SSSLs) from 16 donors and Hua-jing-xian74		
Grain size	*GS3*	Aluko et al., 2004	Doubled haploid BC_3_F_1_ of Caiapo^∗^4/*Oryza glaberrima*	[Bibr B80][Bibr B105]	Association studiesAssociation studies
		Fan et al., 2006	BC_3_F_2_ of Minghui 63^∗^4/Chuan7		
Gelatinization temperature	*SSIIa*	Umemoto et al., 2002	BC_1_F_8_ of Nipponbare^∗^2/Kasalath	Bao et al., 2006	Association studies


		Waters et al., 2006	Seventy rice varieties originating from different countries and breeding lines from Australia	Gao et al., 2011	Map-based cloning, association studies
		Bao et al., 2006	Sequencing analysis of 30 rice varieties, association studies of 509 rice samples	Lu et al., 2010	Association mapping
Iron (Fe)	*OsYSL1*, *OsMTP1*	Anuradha et al., 2012	F_6_ recombinant inbred lines (RILs) derived from the cross Madhukar × Swarna	Have yet to be developed	
	*OsFER1*,*OsFER2*	Gross et al., 2003	*Indica* variety genome from Genomic BLAST		
Zinc (Zn)	*OsARD2OsIRT1OsNAS1OsNAS2*	Anuradha et al., 2012	F_6_ RILs derived from the cross Madhukar × Swarna		
Fe and Zn	*OsNAS* gene family	Johnson et al., 2011	Transformation of Nipponbare		
	*OsNAS3*, *OsNRAMP1*, *Heavy metal ion transport*, *APRT*	Anuradha et al., 2012	F_6_ RILs derived from the cross Madhukar × Swarna		

## Advantages of FMs over Random DNA Markers

The advantage of FMs is that they can be applied to any population; random markers discovered from quantitative trait loci (QTL) mapping might be population specific. Parents from the QTL mapping have different genetic backgrounds, and might not be polymorphic when applied to other populations (Lübberstedt et al., 2005; Miklas et al., 2006; Gupta et al., 2010). In contrast, FMs can be used regardless of the genetic background of the population under study and applied to any population without revalidating markers or the QTL relationship (Gupta et al., 2010).

FMs are developed from functional gene motifs and, therefore, have complete linkage to the desired allele (Andersen and Lübberstedt, 2003). Due to the complete linkage of an FM with the target gene and the absence of recombination between the marker and the gene, the loss of information and the false selection in MAB can be prevented (Ingvardsen et al., 2008). Phenotypic validation in MAB that uses random DNA markers is essential to ensure that the target gene and marker are transferred together to the progeny; however, using FMs eliminates the need for phenotypic validation (Andersen and Lübberstedt, 2003). Therefore, FMs are more efficient than random DNA markers in MAB applications.

Another major concern in MAB is linkage drag. Random DNA markers might be located far from the target genes; therefore, when they are applied in MAB, a larger donor segment will be introgressed into the recipient parent or backcross progeny. Undesirable genes might be transferred along with the target gene, resulting in reduced performance of the phenotypic trait. To minimize linkage drag in MAB, Hospital (2001) suggested the use of flanking markers closely linked to the introgressed gene in a large population size to obtain double-recombinant genotypes. Alternatively, FMs can reduce linkage drag, particularly in foreground selection by genotyping a smaller population size (Bagge and Lübberstedt, 2008; Gupta et al., 2010).

## A Brief Review on the Genetics of Rice Quality

Eating quality refers to the consumers’ sensory perception of cooked rice, which is related to characters such as flavor and texture (Hsu et al., 2014). Cooking quality refers to chemical reactions resulting from the cooking of the grain, such as gelatinization temperature (GT), kernel elongation, and water uptake (Juliano and Perez, 1984; Hsu et al., 2014). Amylose, a constituent of starch which comprises approximately 95% of the grain dry weight (Fitzgerald et al., 2009), is an important determinant of eating and cooking qualities. In addition, amylose content (AC) affects the glycemic index of a diet (Juliano and Goddard, 1986; Miller et al., 1992; Fitzgerald et al., 2011). Amylose is synthesized by granule bound starch synthase 1 (GBSSI) (Smith et al., 1997), which is encoded by the *Waxy* gene. At present, many *Waxy* alleles that correspond to different AC classes have been reported. The five common alleles are *wx*, *Wx^t^*, *Wx^g1^*, *Wx^g2^*, and *Wx^g3^*, which correspond to glutinous, low, intermediate, high I, and high II classes of apparent AC, respectively (Teng et al., 2012). In addition to these common alleles, a rare allele, *Wx^op^*, has been reported by Mikami et al. (2008). The identified alleles have given researchers the ability to develop FMs to develop rice varieties with desired AC by using MAB.

The *Waxy* gene has been reported to affect the gel consistency (GC) and GT of rice (Tan et al., 1999; Wang et al., 2007; Tian et al., 2009). Studies have confirmed that *Waxy* gene affects both AC and GC (Fan et al., 2005; Zhang et al., 2012). Although GT has been reported to be influenced by the *Waxy* gene, a major QTL corresponding to the *alkali degeneration* locus (*alk*) was found to control GT (Tian et al., 2005; Wang et al., 2007). The *starch synthase IIa* gene (*SSIIa*), located at the *alk* locus (Umemoto et al., 2002), is reported to have several functional single nucleotide polymorphisms (SNPs), SNP2, SNP3 (Umemoto and Aoki, 2005; Waters et al., 2006), and SNP4, (Bao et al., 2006; Waters et al., 2006) that affect GT.

Rice grain appearance is an important aspect that affects the visual preference of consumers. A major QTL on chromosome 3 has been found to be responsible for grain length (Aluko et al., 2004; Fan et al., 2006). A comparative sequencing study between short- and long-grain varieties showed that the second exon of the putative grain length gene *GS3* has a nonsense mutation that is found in long-grain varieties (Fan et al., 2006). On the other hand, a loss of function mutation in *GW2*, a QTL located on chromosome 2, affects the grain width and weight (Song et al., 2007).

Fragrant rice varieties, such as basmati and jasmine, are of great interest to consumers due to their distinctive flavor. Researchers have identified many chemical compounds that contribute to the fragrance of fragrant rice (Yajima et al., 1979; Petrov et al., 1996). Of the identified chemical compounds, 2-acetyl-1-pyrroline (2AP) has been found to be the most significant compound in conferring fragrance to fragrant rice (Buttery et al., 1983, 1988). The elevated levels of 2AP in fragrant rice are thought to be due to a deletion within exon 7 (Bradbury et al., 2005a; Amarawathi et al., 2008) or exon 2 (Shi et al., 2008) of the gene encoding the enzyme betaine aldehyde dehydrogenase (BADH2), which is located on chromosome 8. These mutations render BADH2 non-functional, resulting in the accumulation of 2AP (Bradbury et al., 2005a, 2008). However, the deletions within exon 2 and exon 7 are likely not the only mutations for fragrance because there are varieties without them that accumulate 2AP (Fitzgerald et al., 2008). Fitzgerald et al. (2008) suggest that other mutations could influence BADH2 or that there exist other biochemical pathways, such as the one proposed by Huang et al. (2008), in addition to the pathway proposed by Bradbury et al. (2008), that lead to 2AP accumulation. Hence, the genetics and biochemical pathways of fragrance should be investigated by researchers to further understand fragrance in rice.

The micronutrients Zn and Fe are present in low quantities in rice, especially in the polished grain (Mayer et al., 2008). Therefore, biofortification strategies are undertaken to enhance the nutritional quality of rice in order to avert micronutrient deficiencies in populations for whom rice is the staple food and who have limited access to other fortified foods or micronutrient supplements (Bouis and Welch, 2010). However, biofortification in rice is no simple task. Sperotto et al. (2012) stated five constraints for concern in Fe biofortification: uptake from the soil, loading of the xylem, transport through the phloem, unloading at the base of the grain, and grain sink strength. The genetic engineering approach has reported success in increasing Zn and Fe content by overexpression of genes such as *ferritin* and those of the *OsNAS* gene family, which encode proteins that serve different purposes such as Fe accumulation or the transport of Fe ions (Johnson et al., 2011; Paul et al., 2012). Many QTLs for Zn and Fe have been reported, and candidate genes and linked markers have also been identified (Lu et al., 2008; Garcia-Oliveira et al., 2009; Sperotto et al., 2010; Anuradha et al., 2012). Based on specific QTLs, linked markers and candidate genes, the development of FMs for Zn and Fe improvement is anticipated for MAB programs.

## Progression toward FMs for Quality Rice Breeding

Before the era of molecular marker technology, grain quality was evaluated on palatability, and the presence or absence of a certain trait, such as aroma. The evaluation of rice quality can also be performed visually, providing morphological data, which can then be represented by a morphological marker. Scientists investigate the proteins or enzymes underlying a specific trait, known as an allozyme marker, to discover the exact cause of the trait. Due to ambiguity and the limited information that can be extracted from enzyme analysis (Murphy et al., 1996), scientists’ attention has shifted toward DNA markers.

One classic example of DNA markers is the RFLP marker. RFLP is a hybridization based marker that utilizes restriction enzymes to cut the DNA at specific restriction sites. Single nucleotide changes, insertions or deletions cause changes in restriction sites, resulting in different molecular weight restriction fragments and variation between individuals. RFLP markers were used to map genes to chromosomes. Once a RFLP marker has been positively identified as linked to the putative gene controlling the trait under study, further investigation, such as chromosome walking, cloning, or sequencing of the gene, is undertaken. For instance, the gene controlling fragrance was initially mapped by Ahn et al. (1992) using a RFLP marker; using near isogenic lines (NILs), RFLP analysis showed that the fragrance gene (*fgr*) is linked to marker RG28 on chromosome 8.

The introduction of PCR-based markers such as SSR has increased scientists’ knowledge of the genetic map. The locus for a certain trait previously mapped with RFLP is saturated with SSR markers, thereby increasing proximity to the gene. In the case of fragrance, the *fgr* locus was mapped with SSR markers after it was discovered (Chen et al., 1997; Cho et al., 1998). Subsequently, the identified SSR markers have facilitated the development of SSR markers closely linked to the *fgr*, such as that developed by Garland et al. (2000) which detects changes in the mononucleotide repeat of thiamine, (T)_*n*_. This marker was unable to discriminate between genotypes using low-resolution agarose gels and was not polymorphic for some rice variety combinations; therefore, Cordeiro et al. (2002) developed another SSR marker based on the (AT)_40_ repeat for fragrance genotyping.

**FIGURE 1:**
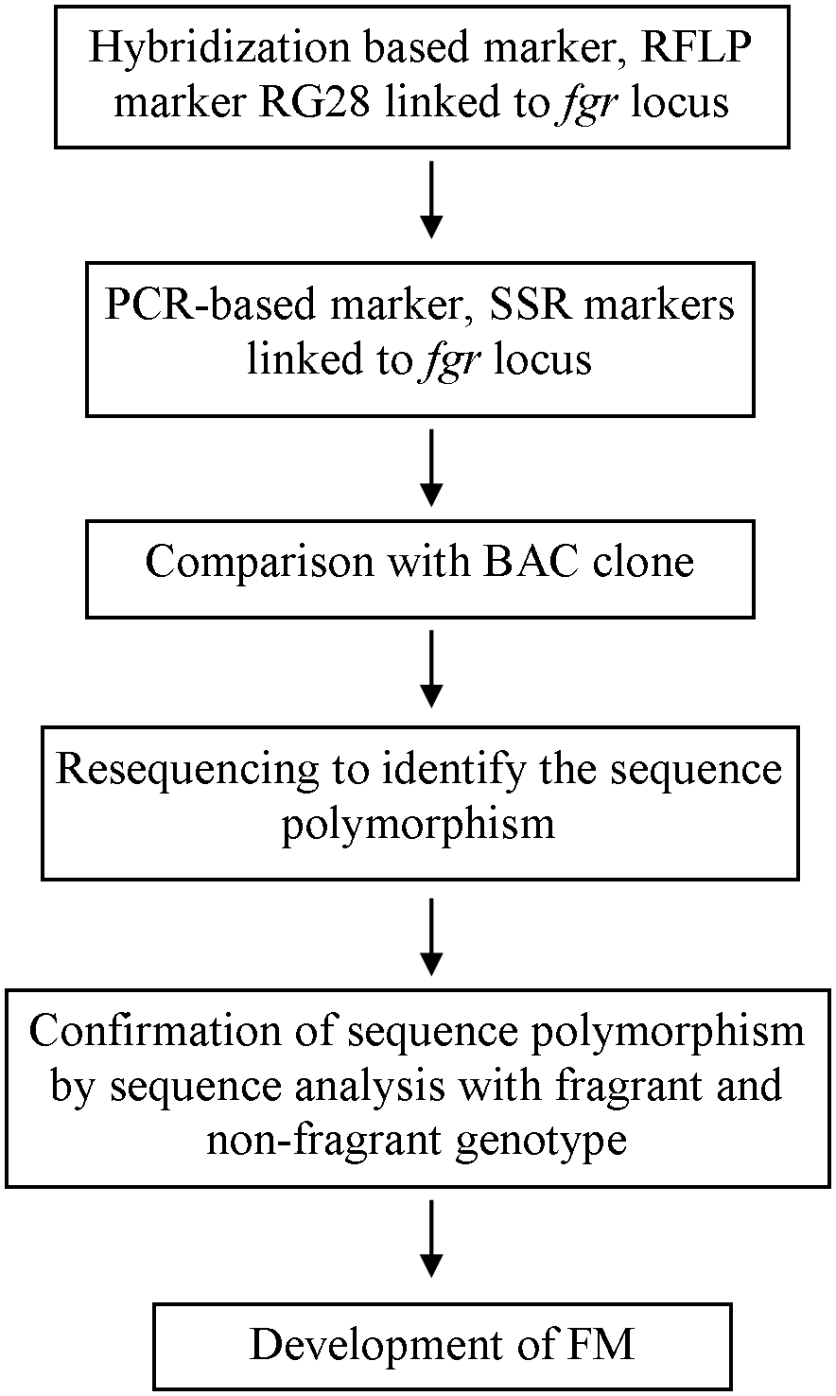
**A chart of simplified progress of functional marker (FM) development for trait used as an example, fragrance, with reference to Ahn et al. (1992) and Bradbury et al. (2005a)**.

Researchers’ efforts to identify linked markers have facilitated further exploration into the genes responsible for rice quality traits. Sequencing the rice genome has also facilitated gene discovery (IRGSP, 2005; 3K RGP, 2014); now that rice genomic sequence data are available, genotype sequences of rice with and without a desirable trait can be compared, leading to discovery of the sequence underlying the trait. Using a linked SSR marker and a bacterial artificial chromosome (BAC), Bradbury et al. (2005a) identified the sequence polymorphism between fragrant and non-fragrant varieties, that is an 8-bp deletion and three SNPs and found the gene (later known as *badh2*) that codes for BADH2 whose functionality determines 2AP accumulation in rice. Based on sequence polymorphism and allele variation studies on different fragrant genotypes, researchers have developed FMs for use in genotyping and breeding (Bradbury et al., 2005b; Amarawathi et al., 2008; Shi et al., 2008; Sakthivel et al., 2009; Vanavichit et al., 2010; Myint et al., 2012). The progress of FM development for the example of fragrance is shown in a simplified manner in **Figure 1**. Some of the FMs developed by researchers for use in quality rice breeding are listed in **Table 1**.

## The Development and Applications of FMs in Quality Rice Breeding

**FIGURE 2:**
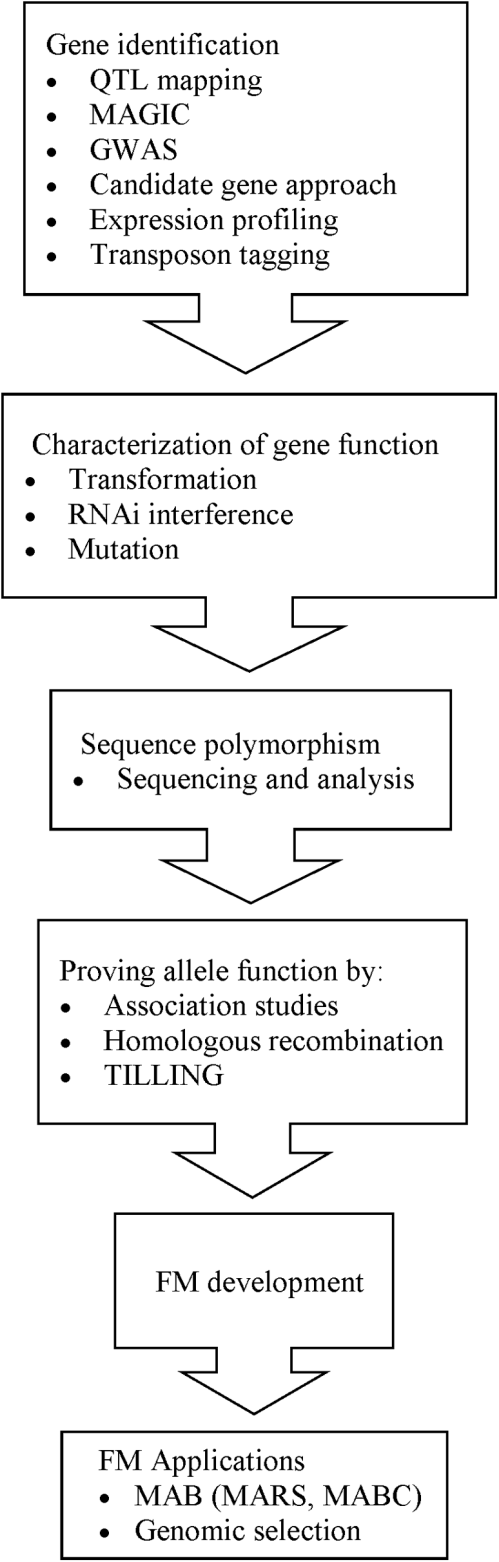
**Flow chart of the development and applications of FMs**.

The development of FMs involves a series of steps (**Figure 2**). The initial step is discovery of the gene that controls the trait. Forward and reverse genetics approaches facilitate the identification of genes that casually affect phenotypic variation. One method of gene identification is by QTL mapping, which identifies loci that underlie the gene or genes that contribute to the trait. Family-based QTL mapping requires the development of a pedigree from crosses between different genotypes and their resulting progeny. Over the years, many family-based QTL mapping studies, especially bi-parental QTL mapping, have been conducted for rice quality traits. However, in family-based QTL mapping, the recombination events are limited to the generations of the family and therefore provide low resolution (Mitchell-Olds, 2010). To improve the resolution of QTL mapping and promote more recombination events, researchers can opt for multiple-parent advanced generation inter-cross (MAGIC). Bandillo et al. (2013) have developed a MAGIC population from half diallel-mating of eight varieties; conducted genotyping by sequencing (GBS) on 200 *indica* MAGIC lines and identified major genes and QTLs for many traits that influence grain quality, such as *Waxy* and *GS3*. Conversely, population-based QTL mapping and genome-wide association studies (GWAS) take into account the historical recombination events that have accumulated over thousands of generations and are, therefore, able to provide higher resolution (Mitchell-Olds, 2010). GWAS utilizes more than 100 genotypes with diverse backgrounds, which leads to a broader genetic base (Mitchell-Olds, 2010). GWAS investigates genome-wide association between SNPs and phenotypes, utilizing an array-based SNP detection platform or next generation sequencing (NGS). Chen et al. (2014) developed an array-based genotyping tool called RiceSNP50 and identified a locus in the same region as the *GS3* locus. Huang et al. (2010) utilized NGS and conducted GWAS on 373 *indica* lines for 14 agronomic traits important to grain quality and identified major genes such as *Waxy* and the *alk* locus, which were similar to those reported by other researchers and other minor genes. Their study showed that GWAS has the potential to identify genes that contribute to natural variation of complex traits. Although the cost for this sequencing platform may be high, with time, it will be made affordable to all researchers. With the marker linked to the loci, target genes can be isolated by map-based cloning (or positional cloning), expression profiling (Duan and Sun, 2005) or transposon tagging, enabling researchers to investigate gene function.

The candidate gene approach has been used in various crop plants to identify genes that contribute to phenotypic variation. Because rice is composed mostly of starch, genes related to starch synthesis are targets of study. Tian et al. (2009) selected 18 starch synthesis-related genes and conducted an association study with AC, GC, and GT. According to Pflieger et al. (2001), genetic transformation is required to determine whether the candidate gene is the gene that causes the trait variation. Tian et al. (2009) have verified the role of each gene in the starch synthesis system by transformation. Their results suggest that selection of a single gene might be insufficient because starch synthesis-related genes cooperate with each other to form a network that determines AC, GC, and GT; therefore, modifying a single gene may alter these three properties. The verified candidate genes from this study can potentially be used in FM development.

Currently, the availability of the rice genome sequence (IRGSP, 2005; 3K RGP, 2014) facilitates gene discovery. Despite this resource, not all genes have had their functions characterized. Well-characterized gene function is a prerequisite of FM development. There are several methods by which researchers can determine a gene’s function, including genetic transformation, RNA interference (RNAi) and mutant characterization. To determine the function of the *OsBADH2* gene, Niu et al. (2008) used RNAi combined with *Agrobacterium tumefaciens*-mediated T-DNA transfer. Their results demonstrated that down-regulated expression of the *OsBADH2* gene resulted in increased 2AP, thereby validating *OsBADH2* as a gene that affects fragrance in rice. Bradbury et al. (2008) proposed a pathway involving BADH2 that leads to 2AP accumulation; this pathway was supported by Chen et al. (2008), who studied it by transformation. A study by Gross et al. (2003) reported the *ferritin* genes *OsFER1* and *OsFER2*. An expression profile study on *OsFER1* and *OsFER2* was then conducted by Stein et al. (2009), who showed that treatment with copper, excess Fe, and other metals causes differential expression of *OsFER1* and *OsFER2*. Paul et al. (2012) showed that overexpression of the*OsFER2* gene led to increased Fe and Zn levels in T_3_ transgenic plants.

Polymorphisms in the alleles that contribute to variation in phenotype can be in the form of SNPs, insertions/deletions (Indels) or SSRs. The relationship between the allelic polymorphism and the phenotypic variation is tested by either indirect or direct proof of allele function (Andersen and Lübberstedt, 2003). Association study is an indirect approach for proving allele function, which provides statistical proof of the relationship between the allele polymorphism and phenotype. Association studies rely on linkage disequilibrium (LD) (Andersen and Lübberstedt, 2003), which plays an important role in association studies because it affects the fine mapping of agronomically important genes. Because rice (*Oryza sativa*) is an autogamous species, the LD of approximately 75 kb for the *indica* variety is considered high; therefore it is eligible for genome-wide LD association mapping (Mather et al., 2007; Zhao et al., 2011).

Alternatively, reverse genetics approaches such as homologous recombination (HR) or targeted induced local lesions in genomes (TILLING) can be used to directly identify motif function. HR is the locus-targeted integration of alleles to produce isogenic genotypes to obtain direct proof of allele function (Andersen and Lübberstedt, 2003; Hanin and Paszkowski, 2003). Research by Terada et al. (2002) used gene targeting by HR to investigate the *Waxy* locus in rice with a positive/negative selection vector; these researchers obtained approximately 1% survival of transformants, suggesting that the method can be useful for gene-targeting or gene-knockout. The effects on the phenotypes of organisms generated from HR can, therefore, provide direct proof of allele function.

Targeted induced local lesions in genomes approach involves mutagenesis to create variations of mutants which are then subject to high-throughput screening for mutation discovery. By using two Nipponbare populations treated with ethyl methanesulphonate (EMS) or a combination of sodium azide andmethyl-nitrosourea (Az-MNU), Till et al. (2007) reported mutation rates of 1/294 kb and 1/265 kb, respectively. Suzuki et al. (2008) reported a mutation rate of 1/135 kb from a Taichung 65 mutant population treated with MNU, suggesting that a high mutation rate can be used to compliment other mutant resources in rice. EcoTILLING, a variant of TILLING, has been effective at revealing allele polymorphism and acts as a useful marker system for resistant genes in barley (Mejlhede et al., 2006). Recently, Tsai et al. (2011) incorporated NGS with multidimensional pooling into a TILLING protocol for identification of rare alleles. This advent in sequencing, along with researchers’ substantial efforts, will lead to the discovery of more alleles. Polymorphisms detected in mutants from TILLING or EcoTILLING provide proof of allele function; when coupled with phenotypic data, these results can facilitate the development of FMs.

Plant breeding has benefited from the advent of marker technology. The application of markers in plant breeding is known as MAB. MAB includes marker-assisted recurrent selection (MARS), marker-assisted backcrossing (MABC), and marker-assisted gene pyramiding. MABC is the most commonly used technique in rice breeding. In foreground selection of MABC, markers associated with the QTL or genes for the desired trait are used to identify plants that carry the preferred allele, allowing selection to be conducted at an early stage of the breeding program. Using an FM rather than a linked DNA marker can improve the selection precision. Because the FM is in complete linkage with the target gene, the risk of linkage drag and recombination between the marker and target gene can be minimized, thereby reducing the chance of undesirable alleles being passed down from the donor parent. Jin et al. (2010) applied FMs to select *Waxy*, *badh2*, and *SSIIa* genes in their backcrossing scheme and successfully improved the AC, GT, and fragrance of a maintainer line used for hybrid rice production. Study by Jin et al. (2010) showed that the availability of FMs has made possible the introgression of three traits simultaneously in a breeding program. Moreover, using FMs saves time as it circumvents the phenotypic evaluation on a limited number of seeds at every stage of breeding that would be conducted in conventional breeding.

Marker-assisted breeding is particularly useful for traits controlled by major QTLs or genes with large effects; however, it may be ineffective for traits governed by many QTLs with small effects or those influenced by the environment. Genomic selection (GS), an alternative to MAB proposed by Meuwissen et al. (2001), utilizes all marker and phenotypic data to estimate marker effects and makes predictions of which individuals would make the best parents. The genomic estimated breeding values (GEBVs) are calculated from a training population, for which both genotypic and phenotypic data have been collected and then tested on the candidate population (Chen et al., 2013). Recently, Spindel et al. (2015) attempted GS on rice for three traits: flowering time, plant height, and grain yield. Their study reported more accurate predictions of breeding line performance than pedigree data alone. With its many strengths, GS is anticipated to aid researchers in breeding for micronutrients such as Fe and Zn where many QTLs or genes are involved.

## Challenges in the Development and Application of FMs in Breeding for Quality Rice

Although a plethora of developed FMs are recommended for application in quality rice breeding programs, researchers who have used them have different experiences and opinions on use. Amarawathi et al. (2008) and Sakthivel et al. (2009) reported inconsistency of the allele-specific amplification (ASA) marker system for fragrant rice genotyping developed by Bradbury et al. (2005a). Conversely, Sarhadi et al. (2011) proved the efficacy and efficiency of ASA markers in differentiating fragrant and non-fragrant rice genotypes and the genotype matches the phenotype accurately. These contrary views suggest that proper optimization of the FM assay prior to its use is essential because an optimized assay ensures reproducible results; therefore, optimization is required prior to its application in breeding programs (Poczai et al., 2013).

Another concern for the application of FMs is the pleiotropic effects of certain genes on several traits (Chen and Lübberstedt, 2010; Brenner et al., 2013). Understanding the correlation among characteristics or the pleiotropy of major genes allows breeders to decide which traits should be directly or indirectly selected or to compensate for the undesirable traits with favorable alleles (Chen and Lübberstedt, 2010). Although major genes or QTLs that influence GT and GC have been identified [*SSIIa* (Umemoto et al., 2002), *alk2(t)* (Shu et al., 2006), and *qGC-6* (Su et al., 2011)], the effect of the *Waxy* gene on GT and GC (Lanceras et al., 2000; Bao et al., 2003; Zhang et al., 2012), has yet to be determined as pleiotropic or gene linkage. This create a challenge to breeders in selecting the traits in breeding programs (Shu et al., 2006).

Epistasis is another concern for breeders because it complicates the inheritance of quality traits. If epistatic effects of the genes are not taken into account, the associations for a single gene might be inaccurate or misleading, thereby hindering the development of FMs and causing inconsistency in FM application (Brenner et al., 2013). Epistatic effects among the QTLs controlling quality traits have been reported (Lee and Koh, 2010; Anuradha et al., 2012; Liu et al., 2013); therefore, researchers should discern the epistatic effects of the genes influencing a trait in quality rice breeding.

The main advantage of an FM is that it would have complete linkage with the desired allele; therefore, it could be applied to any population, regardless of genetic background, without having to revalidate the QTL relationship. While the above statement is technically correct, there is a subtle complication that needs to be mentioned. If a researcher selects on the basis of an FM, the possibility remains that the phenotype of interest is due to another allele that is in linkage disequilibrium with the FM. However, the effect is small. Although no large-scale assessment of linkage disequilibrium has been observed in *O. sativa*, the seminal work by Garris et al. (2003) indicated a linkage disequilibrium decay of 100 kb around a disease resistance locus in the *aus* subpopulation. More recently, linkage disequilibrium decays of 50 kb in *indica*, 5 kb in *Oryza rufipogon* (Rakshit et al., 2007), 2 Mb in *indica* and tropical *japonica*, and 500 kb in *O. rufipogon* have been reported by using gene-based markers and phenotypes (Caicedo et al., 2007). The physical extent of linkage disequilibrium around a gene defines the efficiency of linkage disequilibrium mapping, which is the consequence of several factors, including the degree of artificial or natural selection on the gene or region of the genome, the rate of outcrossing, recombination fraction, the age of the allele under study, chromosomal location, and the population size and structure (Garris et al., 2003).

While the costs of development and establishment and application of FMs in MAB might currently be a concern to researchers, the costs of marker development and marker genotyping are expected to drop in the near future (Lau et al., 2014). Monsanto reported that price per molecular marker decreased over sixfold from 2000 to 2006 (Eathington et al., 2007). Cost for marker discovery by sequencing technology is also expected to decrease over time (Wetterstrand, 2014). Therefore, the advancement of sequencing technology is expediting gene discovery and FM development.

## Conclusion

There are more genes involved in eating, cooking, and the nutritional qualities of rice that have not yet been discovered. Whole-genome sequencing of rice has been conducted to identify some of these genes. The discovery of genes and gene function characterization can be conducted using the various approaches of forward and reverse genetics. FMs could revolutionize the selection strategy in quality rice breeding without linkage drag. Because the cost of marker discovery by sequencing technology is decreasing, the adoption of FMs in breeding programs, especially MAB, is greatly anticipated. We envision that the utilization of FMs will enable the incorporation of all genes for cooking, eating, and nutritional qualities into one rice genotype.

## Conflict of Interest Statement

The authors declare that the research was conducted in the absence of any commercial or financial relationships that could be construed as a potential conflict of interest.

## References

[B1] 3K RGP (2014). The 3,000 rice genomes project. *Gigascience* 3 7 10.1186/2047-217X-3-7 PMC4035669 24872877

[B2] Ahmad R. (2013). *Rice Revolution | Bangladesh Set to Release the World’s First zinc-Enriched Variety. Dly. Star.* Available at: http://archive.thedailystar.net/beta2/news/rice-revolution/ [accessed October 15 2014].

[B3] Ahn S. N. Bollich C. N. Tanksley S. D. (1992). RFLP tagging of a gene for aroma in rice. *Theor. Appl. Genet.* 84 825–828. 10.1007/BF00227391 24201481

[B4] Aluko G. Martinez C. Tohme J. Castano C. Bergman C. Oard J. H. (2004). QTL mapping of grain quality traits from the interspecific cross *Oryza sativa* x *O. glaberrima*. *Theor. Appl. Genet.* 109 630–639. 10.1007/s00122-004-1668-y 15105992

[B5] Amarawathi Y. Singh R. Singh A. K. Singh V. P. Mohapatra T. Sharma T. R. (2008). Mapping of quantitative trait loci for basmati quality traits in rice (*Oryza sativa* L.). *Mol. Breed.* 21 49–65. 10.1007/s11032-007-9108-8

[B6] Andersen J. R. Lübberstedt T. (2003). Functional markers in plants. *Trends Plant Sci.* 8 554–560. 10.1016/j.tplants.2003.09.010 14607101

[B7] Anuradha K. Agarwal S. Rao Y. V. Rao K. V. Viraktamath B. C. Sarla N. (2012). Mapping QTLs and candidate genes for iron and zinc concentrations in unpolished rice of Madhukar × Swarna RILs. *Gene* 508 233–240. 10.1016/j.gene.2012.07.054 22964359

[B8] Ayres N. M. McClung A. M. Larkin P. D. Bligh H. F. J. Jones C. A. Park W. D. (1997). Microsatellites and a single-nucleotide polymorphism differentiate apparent amylose classes in an extended pedigree of US rice germ plasm. *Theor. Appl. Genet.* 94 773–781. 10.1007/s001220050477

[B9] Bagge M. Lübberstedt T. (2008). Functional markers in wheat: technical and economic aspects. *Mol. Breed.* 22 319–328. 10.1007/s11032-008-9190-6

[B10] Bandillo N. Raghavan C. Muyco P. A. Sevilla M. A. L. Lobina I. T. Dilla-Ermita C. J. (2013). Multi-parent advanced generation inter-cross (MAGIC) populations in rice: progress and potential for genetics research and breeding. *Rice* 6 11 10.1186/1939-8433-6-11 PMC4883706 24280183

[B11] Bao J. Corke H. He P. Zhu L. (2003). Analysis of quantitative trait loci for starch properties of rice based on an RIL population. *Acta Bot. Sin.* 45 986–994.

[B12] Bao J. S. Corke H. Sun M. (2006). Nucleotide diversity in starch synthase IIa and validation of single nucleotide polymorphisms in relation to starch gelatinization temperature and other physicochemical properties in rice (*Oryza sativa* L.). *Theor. Appl. Genet.* 113 1171–1183. 10.1007/s00122-006-0394-z 16850313

[B13] Bouis H. E. Welch R. M. (2010). Biofortification-a sustainable agricultural strategy for reducing micronutrient malnutrition in the global south. *Crop Sci.* 50 S-20–S-32. 10.2135/cropsci2009.09.0531

[B14] Bradbury L. M. T. Fitzgerald T. L. Henry R. J. Jin Q. Waters D. L. E. (2005a). The gene for fragrance in rice. *Plant Biotechnol. J.* 3 363–370. 10.1111/j.1467-7652.2005.00131.x 17129318

[B15] Bradbury L. M. T. Henry R. J. Jin Q. Reinke R. F. Waters D. L. E. (2005b). A perfect marker for fragrance genotyping in rice. *Mol. Breed.* 16 279–283. 10.1007/s11032-005-0776-y

[B16] Bradbury L. M. T. Gillies S. A. Brushett D. J. Waters D. L. E. Henry R. J. (2008). Inactivation of an aminoaldehyde dehydrogenase is responsible for fragrance in rice. *Plant Mol. Biol.* 68 439–449. 10.1007/s11103-008-9381-x 18704694

[B17] Brenner E. A. Beavis W. D. Andersen J. R. Lübberstedt T. (2013). “Prospects and Limitations for development and application of functional markers in plants,” in *Diagnostics in Plant Breeding*, eds Lübberstedt T. Varshney R. K. (Dordrecht: Springer), 329–348.

[B18] Buttery R. G. Ling L. C. Juliano B. Turnbaugh J. G. (1983). Cooked rice aroma and 2-acetyl-1-pyrroline. *J. Agric. Food Chem.* 31 823–826. 10.1021/jf00118a036

[B19] Buttery R. G. Turnbaugh J. G. Ling L. C. (1988). Contribution of volatiles to rice aroma. *J. Agric. Food Chem.* 36 1006–1009. 10.1021/jf00083a025

[B20] Caicedo A. L. Williamson S. H. Hernandez R. D. Boyko A. Fledel-Alon A. York T. L. (2007). Genome-wide patterns of nucleotide polymorphism in domesticated rice. *PLoS Genet.* 3:1745–1756. 10.1371/journal.pgen.0030163 17907810 PMC1994709

[B21] Chen H. He H. Zhou F. Yu H. Deng X. W. (2013). Development of genomics-based genotyping platforms and their applications in rice breeding. *Curr. Opin. Plant Biol.* 16 247–254. 10.1016/j.pbi.2013.04.002 23706659

[B22] Chen H. Xie W. He H. Yu H. Chen W. Li J. (2014). A high-density SNP genotyping array for rice biology and molecular breeding. *Mol. Plant* 7 541–553. 10.1093/mp/sst135 24121292

[B23] Chen M.-H. Fjellstrom R. G. Christensen E. F. Bergman C. J. (2010). Development of three allele-specific codominant rice *Waxy* gene PCR markers suitable for marker-assisted selection of amylose content and paste viscosity. *Mol. Breed.* 26 513–523. 10.1007/s11032-010-9419-z

[B24] Chen S. Yang Y. Shi W. Ji Q. He F. Zhang Z. (2008). Badh2, encoding betaine aldehyde dehydrogenase, inhibits the biosynthesis of 2-acetyl-1-pyrroline, a major component in rice fragrance. *Plant Cell* 20 1850–1861. 10.1105/tpc.108.058917 18599581 PMC2518245

[B25] Chen X. Temnykh S. Xu Y. Cho Y. G. McCouch S. R. (1997). Development of a microsatellite framework map providing genome-wide coverage in rice (*Oryza sativa* L.). *Theor. Appl. Genet.* 95 553–567. 10.1007/s001220050596

[B26] Chen Y. Lübberstedt T. (2010). Molecular basis of trait correlations. *Trends Plant Sci.* 15 454–461. 10.1016/j.tplants.2010.05.004 20542719

[B27] Cho Y. G. McCouch S. R. Kuiper M. Kang M.-R. Pot J. Groenen J. T. M. (1998). Integrated map of AFLP, SSLP and RFLP markers using a recombinant inbred population of rice (*Oryza sativa* L.). *Theor. Appl. Genet.* 97 370–380. 10.1007/s001220050907

[B28] Cordeiro G. M. Christopher M. J. Henry R. J. Reinke R. F. (2002). Identification of microsatellite markers for fragrance in rice by analysis of the rice genome sequence. *Mol. Breed.* 9 245–250. 10.1023/A:1020350725667

[B29] David R. B. (2014). *IRRI’s Iron-rich “Miracle Rice” Ready by Year 2029: Can the Country’s Poor Wait?* Available at: http://www.eaglenews.ph/irris-iron-rich-miracle-rice-ready-by-year-2029-can-the-countrys-poor-wait/ [accessed October 15 2014].

[B30] Dela Cruz N. Khush G. S. (2000). “Rice grain quality evaluation procedures,” in *Aromatic Rices*, eds Singh R. K. Singh U. S. Khush G. S. (New Delhi: Mohan Primlani for Oxford & IBH Publishing Co. Pvt. Ltd), 292.

[B31] Duan M. Sun S. S. M. (2005). Profiling the expression of genes controlling rice grain quality. *Plant Mol. Biol.* 59 165–178. 10.1007/s11103-004-7507-3 16217610

[B32] Eathington S. R. Crosbie T. M. Edwards M. D. Reiter R. S. Bull J. K. (2007). Molecular markers in a commercial breeding program. *Crop Sci.* 47 S–154. 10.2135/cropsci2007.04.0015IPBS

[B33] Fan C. Xing Y. Mao H. Lu T. Han B. Xu C. (2006). *GS3*, a major QTL for grain length and weight and minor QTL for grain width and thickness in rice, encodes a putative transmembrane protein. *Theor. Appl. Genet.* 112 1164–1171. 10.1007/s00122-006-0218-1 16453132

[B34] Fan C. C. Yu X. Q. Xing Y. Z. Xu C. G. Luo L. J. Zhang Q. (2005). The main effects, epistatic effects and environmental interactions of QTLs on the cooking and eating quality of rice in a doubled-haploid line population. *Theor. Appl. Genet.* 110 1445–1452. 10.1007/s00122-005-1975-y 15841361

[B35] Ferrero A. Nguyen N. (2004). Constraints and opportunities for the sustainable development of rice-based production systems in Europe. *FAO Rice Conf.* 43 12–13.

[B36] Fitzgerald M. A. Hamilton N. R. S. Calingacion M. N. Verhoeven H. A. Butardo V. M. (2008). Is there a second fragrance gene in rice? *Plant Biotechnol. J.* 6 416–423. 10.1111/j.1467-7652.2008.00327.x 18331415

[B37] Fitzgerald M. A. McCouch S. R. Hall R. D. (2009). Not just a grain of rice: the quest for quality. *Trends Plant Sci.* 14 133–139. 10.1016/j.tplants.2008.12.004 19230745

[B38] Fitzgerald M. A. Rahman S. Resurreccion A. P. Concepcion J. Daygon V. D. Dipti S. S. (2011). Identification of a major genetic determinant of glycaemic index in rice. *Rice* 4 66–74. 10.1007/s12284-011-9073-z

[B39] Gao L. Zhou M. Chen R. Gao H. Yan Q. Zhou W. (2012). Developing and validating the functional marker of rice *Waxy* gene. *Rice Genomics Genet.* 3 61–65. 10.5376/rgg.2012.03.0010

[B40] Gao Z. Zeng D. Cheng F. Tian Z. Guo L. Su Y. (2011). *ALK*, the key gene for gelatinization temperature, is a modifier gene for gel consistency in rice. *J. Integr. Plant Biol.* 53 756–765. 10.1111/j.1744-7909.2011.01065.x 21711449

[B41] Garcia-Oliveira A. L. Tan L. Fu Y. Sun C. (2009). Genetic identification of quantitative trait loci for contents of mineral nutrients in rice grain. *J. Integr. Plant Biol.* 51 84–92. 10.1111/j.1744-7909.2008.00730.x 19166498

[B42] Garland S. Lewin L. Blakeney A. Reinke R. Henry R. (2000). PCR-based molecular markers for the fragrance gene in rice (*Oryza sativa* L.). *Theor. Appl. Genet.* 101 364–371. 10.1007/s001220051492

[B43] Garris A. J. McCouch S. R. Kresovich S. (2003). Population structure and its effect on haplotype diversity and linkage disequilibrium surrounding the *xa5* locus of rice (*Oryza sativa* L.). *Genetics* 165 759–769. 14573486 10.1093/genetics/165.2.759 PMC1462795

[B44] Gross J. Stein R. J. Fett-Neto A. G. Fett J. P. (2003). Iron homeostasis related genes in rice. *Genet. Mol. Biol.* 26 477–497. 10.1590/S1415-47572003000400012

[B45] Gupta P. K. Kumar J. Mir R. R. Kumar A. (2010). “Marker-assisted selection as a component of conventional plant breeding,” in *Plant Breeding Reviews*, ed. Janick J. (Hoboken, NJ: John Wiley & Sons, Inc), 145–217.

[B46] Hanin M. Paszkowski J. (2003). Plant genome modification by homologous recombination. *Curr. Opin. Plant Biol.* 6 157–162. 10.1016/S1369-5266(03)00016-5 12667873

[B47] Hospital F. (2001). Size of donor chromosome segments around introgressed loci and reduction of linkage drag in marker-assisted backcross programs. *Genetics* 158 1363–1379. 11454782 10.1093/genetics/158.3.1363 PMC1461714

[B48] Hsu Y.-C. Tseng M.-C. Wu Y.-P. Lin M.-Y. Wei F.-J. Hwu K.-K. (2014). Genetic factors responsible for eating and cooking qualities of rice grains in a recombinant inbred population of an inter-subspecific cross. *Mol. Breed.* 34 655–673. 10.1007/s11032-014-0065-8 25076839 PMC4092229

[B49] Huang T.-C. Teng C.-S. Chang J.-L. Chuang H.-S. Ho C.-T. Wu M.-L. (2008). Biosynthetic mechanism of 2-acetyl-1-pyrroline and its relationship with Delta1-pyrroline-5-carboxylic acid and methylglyoxal in aromatic rice (*Oryza sativa* L.) callus. *J. Agric. Food Chem.* 56 7399–7404. 10.1021/jf8011739 18680302

[B50] Huang X. Wei X. Sang T. Zhao Q. Feng Q. Zhao Y. (2010). Genome-wide association studies of 14 agronomic traits in rice landraces. *Nat. Genet.* 42 961–967. 10.1038/ng.695 20972439

[B51] Ingvardsen C. Schejbel B. Lübberstedt T. (2008). “Functional markers in resistance breeding,” in *Progress in Botany*, eds Lüttge U. Beyschlag W. Murata J. (Berlin: Springer-Verlag), 61–87. 10.1007/978-3-540-72954-9_3

[B52] IRGSP (2005). The map-based sequence of the rice genome. *Nature* 436 793–800. 10.1038/nature03895 16100779

[B53] Jin L. Lu Y. Shao Y. Zhang G. Xiao P. Shen S. (2010). Molecular marker assisted selection for improvement of the eating, cooking and sensory quality of rice (*Oryza sativa* L.). *J. Cereal Sci.* 51 159–164. 10.1016/j.jcs.2009.11.007

[B54] Johnson A. A. T. Kyriacou B. Callahan D. L. Carruthers L. Stangoulis J. Lombi E. (2011). Constitutive overexpression of the *OsNAS* gene family reveals single-gene strategies for effective iron- and zinc-biofortification of rice endosperm. *PLoS ONE* 6:e24476 10.1371/journal.pone.0024476 PMC3167849 21915334

[B55] Juliano B. O. Goddard M. S. (1986). Cause of varietal difference in insulin and glucose responses to ingested rice. *Qual. Plant. Plant Foods Hum. Nutr.* 36 35–41. 10.1007/BF01091751

[B56] Juliano B. O. Perez C. M. (1984). Results of a collaborative test on the measurement of grain elongation of milled rice during cooking. *J. Cereal Sci.* 2 281–292. 10.1016/S0733-5210(84)80016-8

[B57] Lanceras J. C. Huang Z. L. Naivikul O. Vanavichit A. Ruanjaichon V. Tragoonrung S. (2000). Mapping of genes for cooking and eating qualities in Thai jasmine rice (KDML105). *DNA Res.* 7 93–101. 10.1093/dnares/7.2.93 10819324

[B58] Lau W. C. P. Latif M. A. Rafii M. Y. Ismail M. R. Puteh A. (2014). Advances to improve the eating and cooking qualities of rice by marker-assisted breeding. *Crit. Rev. Biotechnol.* 1–12. 10.3109/07388551.2014.923987 [Epub ahead of print]. 24937109

[B59] Lee J. Koh H. (2010). Epistatic relationships among genes related to endosperm starch synthesis in rice. *Korean J. Breed. Sci.* 42 151–156.

[B60] Liu Q. Jiang J. Niu F. He Y. Hong D. (2013). QTL analysis for seven quality traits of RIL population in Japonica rice based on three genetic statistical models. *Rice Sci.* 20 31–38. 10.1016/S1672-6308(13)60105-5

[B61] Lu K. Li L. Zheng X. Zhang Z. Mou T. Hu Z. (2008). Quantitative trait loci controlling Cu, Ca, Zn, Mn and Fe content in rice grains. . *J. Genet.* 87 305–310. 10.1007/s12041-008-0049-8 19147920

[B62] Lu Y. Xiao P. Shao Y. Zhang G. Thanyasiriwat T. Bao J. (2010). Development of new markers to genotype the functional SNPs of *SSIIa*, a gene responsible for gelatinization temperature of rice starch. *J. Cereal Sci.* 52 438–443. 10.1016/j.jcs.2010.07.008

[B63] Lübberstedt T. Zein I. Andersen J. R. Wenzel G. Krützfeldt B. Eder J. (2005). Development and application of functional markers in maize. *Euphytica* 146 101–108. 10.1093/dnares/dst039

[B64] Mather K. A. Caicedo A. L. Polato N. R. Olsen K. M. McCouch S. Purugganan M. D. (2007). The extent of linkage disequilibrium in rice (*Oryza sativa* L.). *Genetics* 177 2223–2232. 10.1534/genetics.107.079616 17947413 PMC2219496

[B65] Mayer J. E. Pfeiffer W. H. Beyer P. (2008). Biofortified crops to alleviate micronutrient malnutrition. *Curr. Opin. Plant Biol.* 11 166–170. 10.1016/j.pbi.2008.01.007 18314378

[B66] Mejlhede N. Kyjovska Z. Backes G. Burhenne K. Rasmussen S. K. Jahoor A. (2006). EcoTILLING for the identification of allelic variation in the powdery mildew resistance genes *mlo* and *Mla* of barley. *Plant Breed.* 125 461–467. 10.1111/j.1439-0523.2006.01226.x

[B67] Meuwissen T. H. E. Hayes B. J. Goddard M. E. (2001). Prediction of total genetic value using genome-wide dense marker maps. *Genetics* 157 1819–1829. 11290733 10.1093/genetics/157.4.1819 PMC1461589

[B68] Mikami I. Uwatoko N. Ikeda Y. Yamaguchi J. Hirano H. Y. Suzuki Y. (2008). Allelic diversification at the *wx* locus in landraces of Asian rice. *Theor. Appl. Genet.* 116 979–989. 10.1007/s00122-008-0729-z 18305920

[B69] Miklas P. N. Kelly J. D. Beebe S. E. Blair M. W. (2006). Common bean breeding for resistance against biotic and abiotic stresses: from classical to MAS breeding. *Euphytica* 147 105–131. 10.1007/s10681-006-4600-5

[B70] Miller J. Pang E. Bramall L. (1992). Rice: a high or low glycemic index food? *Am. J. Clin. Nutr.* 56 1034–1036. 1442654 10.1093/ajcn/56.6.1034

[B71] Mitchell-Olds T. (2010). Complex-trait analysis in plants. *Genome Biol.* 11:113. 10.1186/gb-2010-11-4-113 PMC2884532 20409352

[B72] Murphy R. W. Jack W. Sites J. Buth D. G. Haufler C. H. (1996). “Proteins: isozyme electrophoresis,” in *Molecular Systemics*, eds Hillis D. M. Moritz C. Mable B. K. (Sunderland, MA: Sinauer Associates). Available at: http://www.researchgate.net/profile/Barbara_Mable/publication/49252417_Molecular_Systematics/links/0046351ade87c46b56000000.pdf [accessed June 29 2015].

[B73] Myint K. M. Arikit S. Wanchana S. Yoshihashi T. Choowongkomon K. Vanavichit A. (2012). A PCR-based marker for a locus conferring the aroma in Myanmar rice (*Oryza sativa* L.). *Theor. Appl. Genet.* 125 887–896. 10.1007/s00122-012-1880-0 22576235

[B74] Niu X. Tang W. Huang W. Ren G. Wang Q. Luo D. (2008). RNAi-directed downregulation of *OsBADH2* results in aroma (2-acetyl-1-pyrroline) production in rice (*Oryza sativa* L.). *BMC Plant Biol.* 8:100 10.1186/1471-2229-8-100 PMC2588449 18840300

[B75] Paul S. Ali N. Gayen D. Datta S. K. Datta K. (2012). Molecular breeding of *Osfer2* gene to increase iron nutrition in rice grain. *GM Crops Food* 3 310–316. 10.4161/gmcr.22104 22992483

[B76] Petrov M. Danzart M. Gaimpaoli P. Faure J. Richard H. (1996). Rice aroma analysis: discrimination between a scented and a non-scented rice. *Sci. Aliments* 16 347–360.

[B77] Pflieger S. Lefebvre V. Causse M. (2001). The candidate gene approach in plant genetics: a review. *Mol. Breed.* 7 275–291. 10.1023/A:1011605013259

[B78] Poczai P. Varga I. Laos M. Cseh A. Bell N. Valkonen J. P. (2013). Advances in plant gene-targeted and functional markers: a review. *Plant Methods* 9 6 10.1186/1746-4811-9-6 PMC3583794 23406322

[B79] Rakshit S. Rakshit A. Matsumura H. Takahashi Y. Hasegawa Y. Ito A. (2007). Large-scale DNA polymorphism study of *Oryza sativa* and *O. rufipogon* reveals the origin and divergence of Asian rice. *Theor. Appl. Genet.* 114 731–743. 10.1007/s00122-006-0473-1 17219210

[B80] Ramkumar G. Sivaranjani A. K. P. Pandey M. K. Sakthivel K. Shobha Rani N. Sudarshan I. (2010). Development of a PCR-based SNP marker system for effective selection of kernel length and kernel elongation in rice. *Mol. Breed.* 26 735–740. 10.1007/s11032-010-9492-3

[B81] Sakthivel K. Shobha Rani N. Pandey M. K. Sivaranjani A. K. P. Neeraja C. N. Balachandran S. M. (2009). Development of a simple functional marker for fragrance in rice and its validation in Indian Basmati and non-Basmati fragrant rice varieties. *Mol. Breed.* 24 185–190. 10.1007/s11032-009-9283-x

[B82] Sarhadi W. A. Hien N. L. Zanjani M. Yosofzai W. Yoshihashi T. Hirata Y. (2011). Comparative analyses for aroma and agronomic traits of native rice cultivars from Central Asia. *J. Crop Sci. Biotechnol.* 11 17–22.

[B83] Shi W. Yang Y. Chen S. Xu M. (2008). Discovery of a new fragrance allele and the development of functional markers for the breeding of fragrant rice varieties. *Mol. Breed.* 22 185–192. 10.1007/s11032-008-9165-7

[B84] Shu X. Shen S. Bao J. Wu D. Nakamura Y. Shu Q. (2006). Molecular and biochemical analysis of the gelatinization temperature characteristics of rice (*Oryza sativa* L.) Starch granules. *J. Cereal Sci.* 44 40–48. 10.1016/j.jcs.2006.03.001

[B85] Smith A. M. Denyer K. Martin C. (1997). The synthesis of the starch granule. *Annu. Rev. Plant Physiol. Plant Mol. Biol.* 48 67–87. 10.1146/annurev.arplant.48.1.67 15012257

[B86] Song X.-J. Huang W. Shi M. Zhu M.-Z. Lin H.-X. (2007). A QTL for rice grain width and weight encodes a previously unknown RING-type E3 ubiquitin ligase. *Nat. Genet.* 39 623–630. 10.1038/ng2014 17417637

[B87] Sperotto R. A. Boff T. Duarte G. L. Santos L. S. Grusak M. A. Fett J. P. (2010). Identification of putative target genes to manipulate Fe and Zn concentrations in rice grains. *J. Plant Physiol.* 167 1500–1506. 10.1016/j.jplph.2010.05.003 20580124

[B88] Sperotto R. A. Ricachenevsky F. K. Waldow V. D. A. Fett J. P. (2012). Iron biofortification in rice: it’s a long way to the top. *Plant Sci.* 190 24–39. 10.1016/j.plantsci.2012.03.004 22608517

[B89] Spindel J. Begum H. Akdemir D. Virk P. Collard B. Redoña E. (2015). Genomic selection and association mapping in rice (*Oryza sativa*): effect of trait genetic architecture, training population composition, marker number and statistical model on accuracy of rice genomic selection in elite, tropical rice breeding lines. *PLoS Genet.* 11:e1004982 10.1371/journal.pgen.1004982 PMC4334555 25689273

[B90] Stein R. J. Ricachenevsky F. K. Fett J. P. (2009). Differential regulation of the two rice ferritin genes (*OsFER1* and *OsFER2*). *Plant Sci.* 177 563–569. 10.1016/j.plantsci.2009.08.001

[B91] Su Y. Rao Y. Hu S. Yang Y. Gao Z. Zhang G. (2011). Map-based cloning proves *qGC-6*, a major QTL for gel consistency of japonica/indica cross, responds by *Waxy* in rice (*Oryza sativa* L.). *Theor. Appl. Genet.* 123 859–867. 10.1007/s00122-011-1632-6 21698394

[B92] Sumner D. A. Rozelle S. D. Huang J. Lee H. (2001). *The China Market for Rice: Current Status, Recent Trends, and Projections, with Emphasis on the Potential for Imports from the United States and Potential for External Competition with U.S. Rice.* Davis, CA: A Research Study for the USA Rice Federation.

[B93] Suzuki T. Eiguchi M. Kumamaru T. Satoh H. Matsusaka H. Moriguchi K. (2008). MNU-induced mutant pools and high performance TILLING enable finding of any gene mutation in rice. *Mol. Genet. Genomics* 279 213–223. 10.1007/s00438-007-0293-2 17952471

[B94] Tan Y. F. Li J. X. Yu S. B. Xing Y. Z. Xu C. G. Zhang Q. (1999). The three important traits for cooking and eating quality of rice grains are controlled by a single locus in an elite rice hybrid. Shanyou 63. *Theor. Appl. Genet.* 99 642–648. 10.1007/s001220051279 22665200

[B95] Teng B. Zeng R. Wang Y. Liu Z. Zhang Z. Zhu H. (2012). Detection of allelic variation at the *Wx* locus with single-segment substitution lines in rice (*Oryza sativa* L.). *Mol. Breed.* 30 583–595. 10.1007/s11032-011-9647-x

[B96] Terada R. Urawa H. Inagaki Y. Tsugane K. Iida S. (2002). Efficient gene targeting by homologous recombination in rice. *Nat. Biotechnol.* 20 1030–1034. 10.1038/nbt737 12219079

[B97] Tian R. Jiang G.-H. Shen L.-H. Wang L.-Q. He Y.-Q. (2005). Mapping quantitative trait loci underlying the cooking and eating quality of rice using a DH population. *Mol. Breed.* 15 117–124. 10.1007/s11032-004-3270-z

[B98] Tian Z. Qian Q. Liu Q. Yan M. Liu X. Yan C. (2009). Allelic diversities in rice starch biosynthesis lead to a diverse array of rice eating and cooking qualities. *Proc. Natl. Acad. Sci. U.S.A.* 106 21760–21765. 10.1073/pnas.0912396106 20018713 PMC2793318

[B99] Till B. J. Cooper J. Tai T. H. Colowit P. Greene E. A. Henikoff S. (2007). Discovery of chemically induced mutations in rice by TILLING. *BMC Plant Biol.* 7:19 10.1186/1471-2229-7-19 PMC1858691 17428339

[B100] Tsai H. Howell T. Nitcher R. Missirian V. Watson B. Ngo K. J. (2011). Discovery of rare mutations in populations: TILLING by sequencing. *Plant Physiol.* 156 1257–1268. 10.1104/pp.110.169748 21531898 PMC3135940

[B101] Umemoto T. Aoki N. (2005). Single-nucleotide polymorphisms in rice starch synthase IIa that alter starch gelatinisation and starch association of the enzyme. *Funct. Plant Biol.* 32 763–768. 10.1071/FP04214 32689173

[B102] Umemoto T. Yano M. Satoh H. Shomura A. Nakamura Y. (2002). Mapping of a gene responsible for the difference in amylopectin structure between japonica-type and indica-type rice varieties. *Theor. Appl. Genet.* 104 1–8. 10.1007/s001220200000 12579422

[B103] Vanavichit A. Tragoorung S. Toojinda T. Wanchana S. Kamolsukyunyong W. (2010). BADH2 nucleic acids associated with grain aroma. U.S. Patent No. 6. Available at: http://www.google.com/patents/US7847083 [accessed September 21 2014].

[B104] Varshney R. K. Graner A. Sorrells M. E. (2005). Genomics-assisted breeding for crop improvement. *Trends Plant Sci.* 10 621–630. 10.1016/j.tplants.2005.10.004 16290213

[B105] Wang C. Chen S. Yu S. (2011). Functional markers developed from multiple loci in *GS3* for fine marker-assisted selection of grain length in rice. *Theor. Appl. Genet.* 122 905–913. 10.1007/s00122-010-1497-0 21107518

[B106] Wang L. Q. Liu W. J. Xu Y. He Y. Q. Luo L. J. Xing Y. Z. (2007). Genetic basis of 17 traits and viscosity parameters characterizing the eating and cooking quality of rice grain. *Theor. Appl. Genet.* 115 463–476. 10.1007/s00122-007-0580-7 17593343

[B107] Waters D. L. E. Henry R. J. Reinke R. F. Fitzgerald M. A. (2006). Gelatinization temperature of rice explained by polymorphisms in starch synthase. *Plant Biotechnol. J.* 4 115–122. 10.1111/j.1467-7652.2005.00162.x 17177790

[B108] Wetterstrand K. (2014). *DNA Sequencing Costs: Data from the NHGRI Genome Sequencing Program (GSP).* Available at: http://www.genome.gov/sequencingcosts/ [accessed January 16 2015].

[B109] World Health Organization (2007). *Preventing and Controlling Micronutrient Deficiencies in Populations Affected by an Emergency.* Geneva: Joint Statement by the World Health Organization, the World Food Programme and the United Nations Children’s Fund. Available at: http://www.who.int/nutrition/publications/WHO_WFP_UNICEFstatement.pdf [accessed March 17 2015].

[B110] Yajima I. Yanai T. Nakamura M. Sakakibara H. Hayashi K. (1979). Volatile flavor components of cooked Kaorimai (scented rice. *O. sativa* japonica). *Agric. Biol. Chem.* 43 2425–2429. 10.1271/bbb1961.43.2425

[B111] Yi M. Than K. Vanavichit A. Chai-arree W. Toojinda T. (2009). Marker assisted backcross breeding to improve cooking quality traits in Myanmar rice cultivar Manawthukha. *Field Crop. Res.* 113 178–186. 10.1016/j.fcr.2009.05.006

[B112] Zhang Z. Li M. Fang Y. Liu F. Lu Y. Meng Q. (2012). Diversification of the *Waxy* gene is closely related to variations in rice eating and cooking quality. *Plant Mol. Biol. Report.* 30 462–469. 10.1007/s11105-011-0362-x

[B113] Zhao K. Tung C.-W. Eizenga G. C. Wright M. H. Ali M. L. Price A. H. (2011). Genome-wide association mapping reveals a rich genetic architecture of complex traits in *Oryza sativa*. *Nat. Commun.* 2 467 10.1038/ncomms1467 PMC3195253 21915109

